# Impact of Gyroscope Integration, Sensor Placement, and Activity Granularity on Human Activity Recognition Performance

**DOI:** 10.3390/s26123683

**Published:** 2026-06-09

**Authors:** Alejandro Castellanos, Antonio M. López, Miguel Á. Salinas, Juan C. Álvarez, Diego Álvarez, Gonzalo García, Ángel Buendía-Romero, Asier Mañas, Raquel Bailón, Vicente Martín, Ana Carbonell-Baeza, Verónica Cabanas-Sánchez, David Martinez-Gomez

**Affiliations:** 1 Multisensor Systems and Robotics Research Group (SiMuR), Electrical Engineering Department, University of Oviedo, 33204 Gijón, Spain; amlopez@uniovi.es (A.M.L.); salinasmiguel@uniovi.es (M.Á.S.); juan@uniovi.es (J.C.Á.); dalvarez@uniovi.es (D.Á.); garciagonzalo@uniovi.es (G.G.); 2 GENUD Toledo Research Group, Faculty of Sports Sciences, Universidad de Castilla-La Mancha, 45071 Toledo, Spain; angel.buendiaromero@uclm.es (Á.B.-R.); asier.manas@ddi.uhu.es (A.M.); 3 CIBER on Frailty and Healthy Aging (CIBERFES), Instituto de Salud Carlos III, 28029 Madrid, Spain; 4 Grupo Mixto de Fragilidad y Envejecimiento Exitoso UCLM-SESCAM, Universidad de Castilla-La Mancha-Servicio de Salud de Castilla-La Mancha, IDISCAM, 45004 Toledo, Spain; 5 Faculty of Education, Psychology and Sport Sciences, University of Huelva, 21007 Huelva, Spain; 6 Biomedical Signal Interpretation and Computational Simulation Research Group (BSICoS), University of Zaragoza, Campus Río Ebro, I+D+i Building—C/Mariano Esquillor, S/N, 50018 Zaragoza, Spain; rbailon@unizar.es; 7 Gene-Environment-Health Interactions Research Group (GIIGAS), Department of Biomedical Sciences, Faculty of Veterinary Science, University of León, 24071 León, Spain; vmars@unileon.es; 8 CTS-1038 eMpOwering health by physical actiVity, Exercise and nutrITion Research Group (MOVE-IT), Department of Physical Education, Instituto de Investigación e Innovación Biomédica de Cádiz (INiBICA), Universidad de Cádiz, 11002 Cadiz, Spain; ana.carbonell@uca.es; 9 Department of Preventive Medicine and Public Health, School of Medicine, Universidad Autonoma de Madrid, 28029 Madrid, Spain; veronica.cabanas@uam.es (V.C.-S.); d.martinez@uam.es (D.M.-G.); 10 CIBER of Epidemiology and Public Health, 28029 Madrid, Spain; 11 IMDEA Nutrition, CEI UAM+CSIC, 28049 Madrid, Spain

**Keywords:** human activity recognition, inertial measurement unit, convolutional neural networks, decision tree-based models, physical activity, machine learning

## Abstract

This study systematically evaluates the impact of sensor configuration, body location, classification granularity, and model choice on inertial-based human activity recognition in a laboratory dataset aligned with the Spanish IMPaCT cohort design. Data were collected from 85 participants instrumented with thigh-, wrist-, and hip-mounted inertial measurement units over a structured protocol of 13 semi-structured daily activities, a resting phase and a structured activity. After manual correction of timestamp drift, signals were segmented into overlapping 10-s windows and analyzed using convolutional neural networks, Random Forest, and XGBoost classifiers.Two classification targets were defined: fine-grained recognition of 15 laboratory-controlled activities and coarse-grained classification into four MET-based intensity levels. Results showed that classification granularity is the primary determinant of performance (F=224.85, *p*-value = $2.304\times 10^{-13}$ through the analysis of variance of the F1-score), with intensity-level classification substantially outperforming fine-grained activity recognition. Sensor configuration, model type, and body location also significantly influenced classification outcomes. Wrist-mounted sensors achieved the highest overall F1-scores. Incorporating gyroscope-derived features consistently improved performance across configurations, and feature importance analysis confirmed their substantial contribution. These findings, derived from models developed under controlled laboratory conditions, provide practical guidance for the design of wearable sensing protocols and modeling strategies in large-scale population-based studies, and support their extension to everyday physical activity, laying the foundation for future real-world applications.

## 1. Introduction

Over the past decade, large-scale national population cohorts have emerged as cornerstone resources for advancing Precision Medicine. The integration of genetic, clinical, behavioral, and lifestyle data from deeply phenotyped populations has enabled new insights and more comprehensive modeling of disease risk, progression, and prevention at the population level. Landmark examples include the All of Us Research Program in the United States [1], the UK Biobank [2,3], and the German National Cohort (NAKO) [4], each of which has established methodological and technological benchmarks for large-scale biomedical research.

Within this framework, objective assessment of physical activity and sedentary behavior has become a methodological priority, given their central role as modifiable risk factors for cardiometabolic, musculoskeletal, and mental health outcomes. Recent cohorts have therefore incorporated triaxial accelerometer-based wearable devices to obtain high-resolution, continuous measurements of movement, typically from a single anatomical location (e.g., wrist, hip, thigh, or ankle). While accelerometry has become the predominant method for large-scale physical activity monitoring, systematic and paired comparative analyses evaluating different sensor placements and complementary sensing modalities under equivalent experimental conditions remain scarce. In particular, the potential contribution of angular velocity signals from gyroscopes—now widely available in modern inertial measurement units (IMUs)—has not been comprehensively examined within population-oriented research designs. The relevance of this research gap is underscored by emerging next-generation initiatives, such as the Spanish IMPaCT cohort [5] (https://cohorte-impact.es), promoted by the Spanish Institute of Health. This large-scale study was designed to recruit 200,000 participants and incorporates the use of wearable devices or wearable sensors equipped with both triaxial accelerometers and gyroscopes, deployed at two body locations: the non-dominant wrist and the contralateral thigh.

Thus, the present study addresses this methodological need using data from the WearablePerMed project. The dataset comprises laboratory and free-living activity recordings from more than 80 participants and includes wearable sensor data collected at the wrist and thigh, in alignment with the IMPaCT design, as well as an additional hip-mounted wearable sensor incorporated as a complementary reference for ongoing methodological developments.

Based on this dataset, we conducted a comprehensive experimental evaluation of activity classification performance across the three sensor locations, explicitly quantifying the contribution of gyroscope data under single-sensor configurations. Deep learning models based on convolutional neural networks were compared with established traditional machine learning approaches, including Balanced Random Forest and XGBoost classifiers. For each modeling strategy, performance was assessed using accelerometer-only signals and combined accelerometer–gyroscope data, enabling a direct estimation of classification improvements attributable to angular velocity measurements. Within this framework, activity recognition was evaluated at two levels of granularity: (i) fine-grained classification of the 15 labeled activity categories included in our dataset, and (ii) coarser classification into four physical activity intensity levels (i.e., sedentary, light, moderate, and vigorous), following established intensity-based taxonomies [6].

## 2. Materials and Methods

### 2.1. Dataset

The employed dataset comprises data from 85 participants (44 women) aged 18 to 75 years from the Madrid and Toledo regions. Linear acceleration and angular velocity signals were acquired from MATRIX 003 wearable sensors (Beijing XMatrix Tech. Co., Ltd., Beijing, China), placed on the dominant wrist, contralateral thigh, and hip.

These wearable sensors integrate a triaxial accelerometer and a triaxial gyroscope, along with additional physiological sensors (i.e., PPG, body and environmental temperatures). The movement sensors operate with a full-scale range of ±16 g and a resolution of 16 bits. Previous studies have reported that the accelerometer noise level is below 3 mg, while the gyroscope exhibits a higher noise level of approximately 0.12 °/s [7,8].

On the first day of the experimental protocol, participants completed an initial resting phase (first activity) lasting 30 min, and a structured incremental cycle ergometer test (fourth activity). For the cycle-ergometer, after a 3-min rest period and a 3-min warm-up (20 W for women and 30 W for men), the workload was increased by 3 W and 4 W every 12 s, respectively, until voluntary exhaustion or until participants were unable to maintain the required pedalling frequency (<60 rpm).

Two additional laboratory activities included in the original protocol in the first day (a sit-to-stand test and a treadmill test) were not considered in the present analysis. The sit-to-stand test was excluded because it represents a specific functional assessment test whose occurrence in free-living conditions is not expected. The treadmill test was excluded because it contained walking and running periods with unidentified transitions at the time of data collection, which hindered reliable labeling of signal windows.

Then, after seven consecutive days of free-living activity monitoring, participants returned to the laboratory on the final day to perform a series of semi-structured activities. Each of these activities lasted exactly 2 min and was carried out in a predefined order. Transitions between activities were natural: participants completed each task and prepared for the next without performing intermediate actions. The order of these activities was: Sitting while watching TV, Sitting while reading, Sitting while using a PC, Standing while using a PC, Standing while folding towels, Standing while moving books, Standing while sweeping, Walking at usual speed, Walking while using a phone or reading a book, Walking while carrying groceries, Zigzag walking, Running at a self-selected speed (20 m walkway), and Stair ascent and descent.

**Figure 1:**
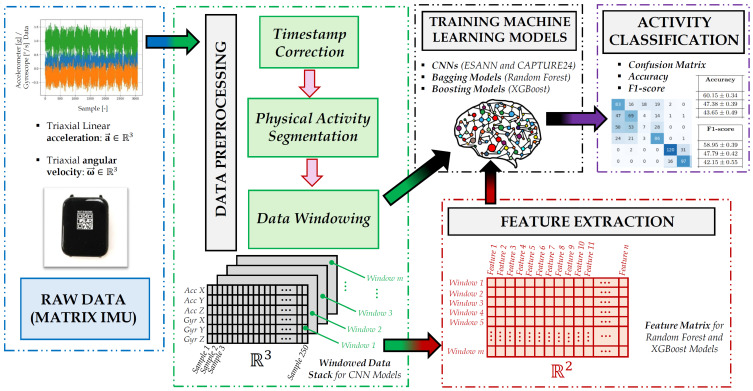
Overview of the methodology employed in this work.

**Figure 2:**
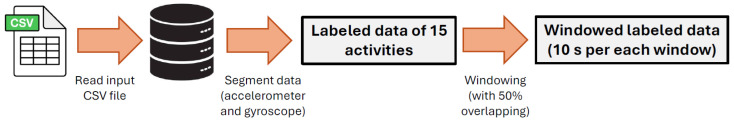
Workflow during data preprocessing.

During pre-processing (see Figure 1 and Figure 2), conducted independently by three researchers, data files from nine participants were discarded due to irreparable errors (for example, damaged BIN accelerometry data files from the MATRIX 003 wearable sensor). Sixteen participants were classified as fully valid, as no errors were identified in their data, while 43 participants had recoverable errors (e.g., filename errors or some recoverable data not registered in experimental annotation forms) that were successfully corrected during the data curation stage, resulting in complete accelerometry datasets. For the remaining 17 participants, valid wearable sensor signals could not be recovered from all three sensors’ locations; nevertheless, partial data were available and sufficient for inclusion in the training of specific classification models. Consequently, wearable data were available for 67 subjects at the thigh, 63 subjects at the wrist, and 63 subjects at the hip.

### 2.2. Data Preprocessing

#### 2.2.1. Timestamp Correction, Activity Segmentation and Signal Windowing

**Figure 3:**
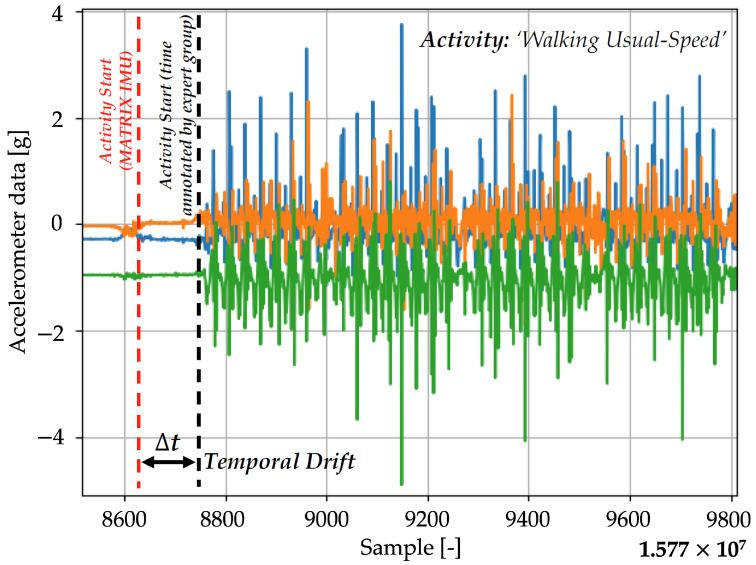
Accelerometry from the thigh (three components *x*, *y*, *z*) of one participant, extracted from the wearable sensor stream using the laboratory log–annotated start time for this activity. A flat segment can be observed on the left side, which does not correspond to the actual activity. The true onset of the activity is indicated by the beginning of oscillatory patterns in the signal. This illustrates the effect of drift, which was corrected linearly by comparing the observed start time in the signal with the start time recorded in the laboratory log, and this correction was subsequently applied to adjust all timestamps recorded by the wearable sensor.

The MATRIX wearable sensor signals were sampled at 25 Hz, recording a UNIX timestamp alongside the internal device signals, including accelerations and angular velocities. Prior to deployment on each participant, all wearable sensors were synchronized using the laboratory computer to establish a common time reference across devices. During the experiments, and according to the data-collection protocol, start and end times of each activity were annotated in a laboratory log using the computer’s clock to enable accurate identification, segmentation, and labeling of the corresponding wearable sensor signal segments for the different activities within the single, continuous activity stream recorded over the seven-day period. No further alignment between the wearable sensors and the computer was performed. As data collection spanned seven days, clock drift in the internal wearable sensor timers could lead to inaccuracies when locating the portions of the signal corresponding to the different laboratory activities based solely on the computer-recorded times registered in the laboratory log (see Figure 3).

To correct for this drift, three researchers manually inspected all signals and identified distinct landmarks corresponding to the onset of specific activities in the accelerometer data, most commonly walking at usual speed, which follows a rest period, or running at self-selected speed (20 m walkway), which produces higher-amplitude signals across all three wearable sensors. The visual inspection of the signals was conducted by consensus between two researchers and supervised by a third researcher, ensuring consistency in event identification. The difference between the device-recorded timestamps at these landmarks and the activity start times from the laboratory log was used to compute a linear correction factor, which was then applied to adjust all wearable sensor timestamps.

Using the corrected timestamps, signal segments corresponding to each laboratory activity were extracted and labeled. The segmented data were subsequently processed using 10-s windows with 50% overlap, applied to both accelerometer and angular velocity signals [6]. Signals were processed as acquired, without applying any normalization or correction for sensor laterality (i.e., left/right placement). In total, the experiments were carried out with 22,862 windows for the thigh location, 21,505 windows for the wrist, and 21,482 windows for the hip.

#### 2.2.2. Classification Targets

Two prediction targets were defined for building the classification models. For fine-grained activity recognition, each of the 15 laboratory activities was treated as an individual class. For coarse grained classification (i.e., physical activity intensity levels), four broader superclasses were defined based on their MET values (see Table 1): Sedentary, Light-Intensity (LI), Moderate-Intensity (MI), and Vigorous-intensity (VI) [6]. This categorization follows the Compendium of Physical Activities [9,10]. Table 1 establishes the mapping between the 15 laboratory activities and the aggregated 4-class intensity categories, used for this coarse-grained classification target.

#### 2.2.3. Feature Selection and Extraction

Windowed signals obtained after signal segmentation (see Figure 1) were used as input features for convolutional neural network classification models. However, Random Forest and XGBoost models require the construction of a feature row vector for each signal window.

The selected features have been used in previous studies on physical activity assessment using accelerometry [6] and applied in population-based cohorts similar to IMPaCT, such as UK Biobank. In this case, the selected and calculated features are as follows:

- Quantiles: minimum, maximum, median, 25th percentile, and 75th percentile of each signal in the signal window.
- Correlation between the accelerometer and gyroscope axes (only used for models incorporating gyroscope data).
- Autocorrelation (with a lag of 1 s) of the accelerometer magnitudes.
- Spectral features: first and second harmonics along with their power, as well as spectral entropy of each signal in the signal window.
- Peak characteristics: number of peaks and average prominence of peaks in each signal within the signal window.
- Angular features: mean of the roll, pitch, and yaw components of the movement. The yaw component has only been taken into account for those models including gyroscope information.

As a result, the feature matrix used to train the Random Forest and XGBoost classifiers has dimensions $R^{m\times 91}$ when all six information channels (triaxial accelerometer and triaxial gyroscope) were included, where *m* denotes the number of training examples available for the classifier. When only accelerometer data were considered, the feature matrix was reduced to $R^{m\times 42}$, reflecting the exclusion of gyroscope-derived features.

### 2.3. Classification Models

Three families of classification models were evaluated: convolutional neural networks (CNNs), Random Forest, and XGBoost, enabling a comparison between deep learning and established ensemble-based machine learning approaches. For the CNN-based models, two architectures were implemented: CNN ESANN, previously applied to public human activity recognition datasets [11], and CNN CAPTURE-24, adapted to our dataset following the methodology described in the CAPTURE-24 study [6].

For all model families, the dataset was partitioned at the participant level to ensure that no subject contributed data to more than one subset. For CNN and XGBoost models, the data were split into training (70%), validation (20%), and test (10%) subsets, while for Random Forest models an 80–20% training–test split was used. To improve robustness and reduce dependency on a single random partition, this splitting procedure was repeated 30 times. In each iteration, participants were randomly shuffled and reassigned to the corresponding subsets, generating 30 independent data splits.

Performance metrics were computed across these 30 repetitions to provide a robust and reliable estimate of model performance.

Hyperparameter optimization for all model families was performed using the Asynchronous Successive Halving Algorithm (ASHA), implemented through Ray Tune [12]; this strategy enables efficient exploration of large hyperparameter spaces while allocating computational resources preferentially to promising configurations. Each experiment was defined by a unique combination of: (i) sensor type (accelerometer-only versus accelerometer + gyroscope), (ii) classification granularity (15 laboratory activities versus four activity intensity levels), (iii) sensor location (thigh, wrist, or hip), and (iv) classification model (CNN ESANN, CNN CAPTURE-24, Random Forest, or XGBoost). For each model, training was repeated 30 times using the optimal hyperparameters identified during tuning. To ensure robust evaluation, the training, validation, and test sets were composed of data from distinct participants.

### 2.4. Feature Importance

To quantify the contribution of gyroscope-derived information, a feature importance analysis was conducted using a Random Forest classifier trained to discriminate the 15 fine-grained activity classes. The analysis was performed independently for each sensor location (thigh, wrist, and hip) using the combined accelerometer–gyroscope feature set. For each configuration, 30 independent training iterations were performed, and the resulting feature importance values were averaged. Feature importance was computed based on the normalized contribution of each feature to the total reduction of impurity across all trees in the Random Forest. Features with an average importance exceeding 1% of the total impurity reduction were considered relevant. This threshold was selected to focus on descriptors with a non-negligible contribution to classification performance.

## 3. Results

**Figure 4:**
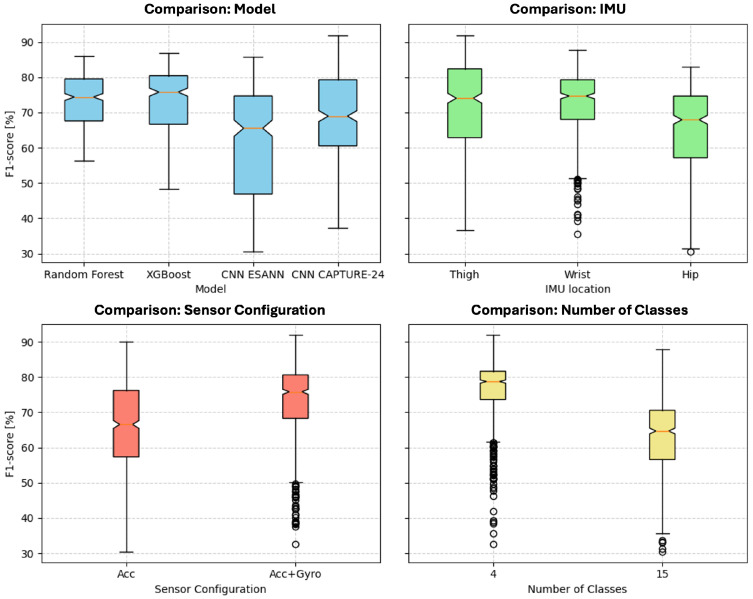
Comparisons of F1-score for the four experimental factors: Model, wearable sensor location, Information used, and Number of Classes. The plots show differences in the F1-score (test) between experimental factors. It should be noted that the MET (Metabolic Equivalent of Task) values, taken from the “Compendium of Physical Activities” (CPA) [9,10], have been used to create the superclasses that categorise the 15 daily activities into 4 levels of physical intensity, in accordance with the mapping shown in Table 1. Consequently, in the lower right subfigure, level 4 associated with the experimental factor “Number of Classes” comprises four physical activity categories: sedentary (Sed.), light-intensity (LI), moderate-intensity (MI) and vigorous-intensity (VI).

**Figure 5:**
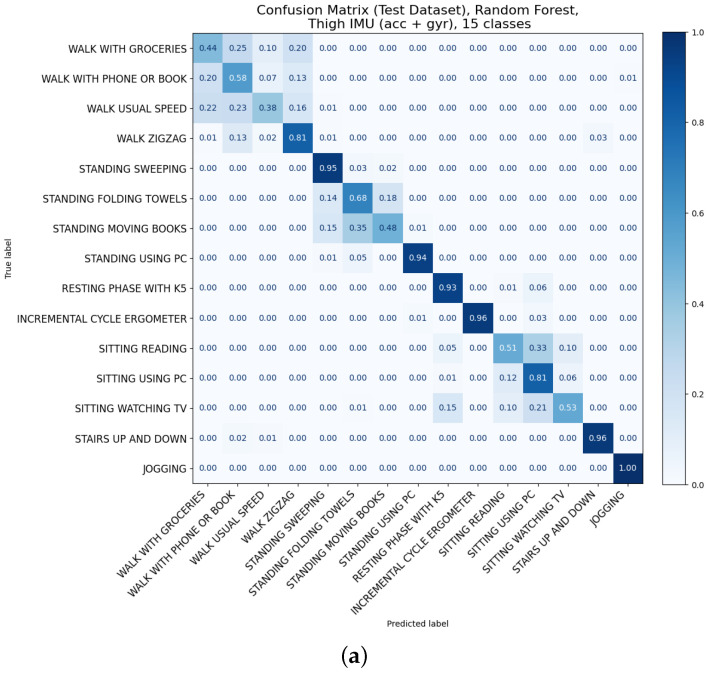

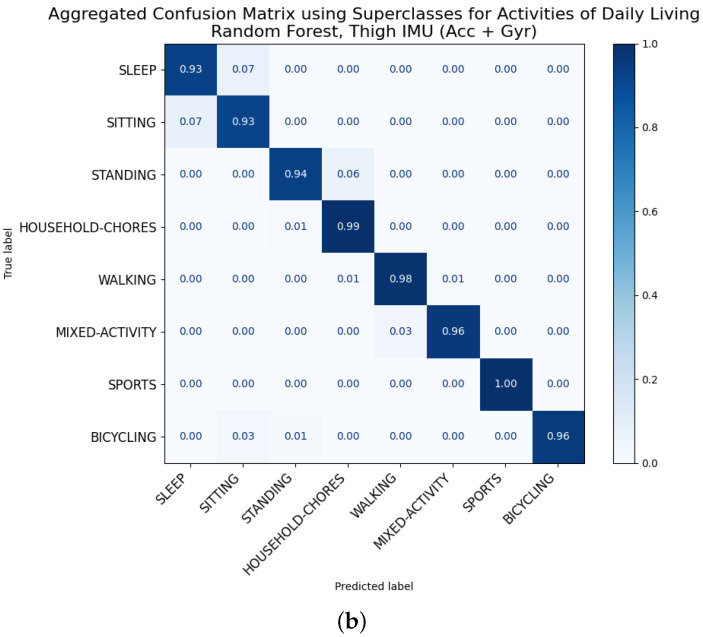
An initial approach to optimising the definition of eight superclasses of daily activities. Both matrices show normalized proportions (values between 0 and 1) obtained with a Random Forest classifier using a thigh-mounted wearable sensor (accelerometer + gyroscope): (**a**) Original confusion matrix in which the model learns a decision boundary capable of distinguishing between 15 categories of daily physical activity; (**b**) Aggregated confusion matrix using 8 superclasses for Activities of Daily Living. This confusion matrix represents an exploratory analysis in terms of the eight superclasses of daily activities.

**Figure 6:**
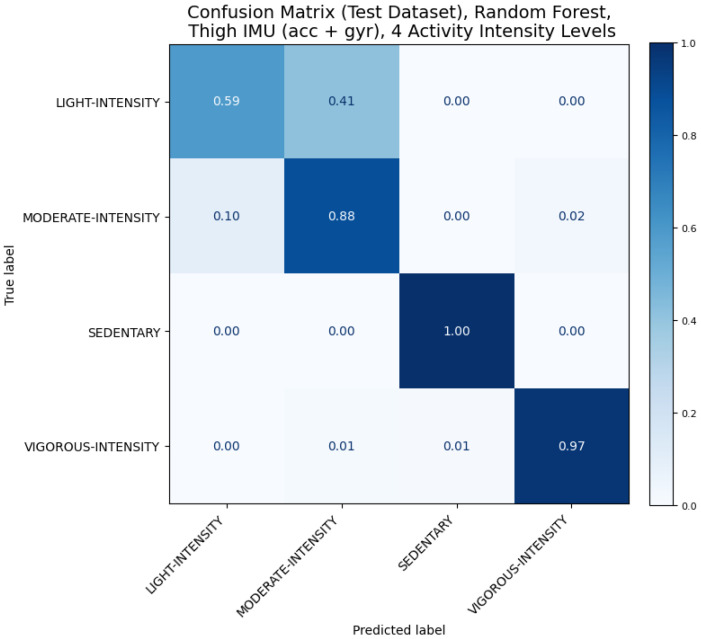
Normalised confusion matrix obtained using a Random Forest model with a thigh-mounted wearable sensor (accelerometer + gyroscope). The decision boundary learned by the model enables the distinction between four levels of physical activity intensity: sedentary, light-intensity, moderate-intensity and vigorous-intensity.

Mean F1-score across 30 independent runs is reported and the corresponding standard deviation (classification performance) on the held-out test dataset, stratified by the combinations of classification model, wearable sensor location, classification granularity, and sensor configuration, are summarized in Table 2. Performance metrics are reported as averages over 30 independent participant-level splits, providing a robust estimate of model generalisation across a heterogeneous population. Overall, classification performance was markedly higher (see Figure 4) for the four activity intensity levels than for the 15-class activity recognition task (see Figure 5a) across all models and sensor locations. The highest performance was achieved by CNN CAPTURE-24 using accelerometer + gyroscope data at the thigh for intensity classification (86.60±2.33), followed by XGBoost (83.52±2.01) and Random Forest (83.46±1.54) under the same configuration. For fine-grained (15-class) recognition, the wrist consistently yielded the best results, with CNN CAPTURE-24 (76.80±5.82), XGBoost (75.45±3.57), and Random Forest (75.38±2.60) showing comparable performance when gyroscope data were included. Incorporating gyroscope signals consistently improved F1-scores across nearly all configurations, with particularly pronounced gains in CNN ESANN at the wrist (from 49.11±5.51 to 70.94±4.12) and hip (from 40.69±5.41 to 60.98±5.76) for the 15-class activities. In general, the wrist location provided the strongest performance for fine-grained activity recognition, whereas the thigh location achieved the highest scores for intensity classification (see Figure 6).

Table 3 summarizes the multifactorial ANOVA assessing the contribution of classification model, IMU location, sensor configuration, and number of classes to the variability in F1-score. The number of classes emerges as the dominant factor (F = 224.85, *p*-value = $2.304\times 10^{-13}$), confirming that task granularity (15 laboratory-controlled activities and 4 intensity levels) exerts the strongest influence on performance. Sensor configuration is the second most influential factor (F = 80.03, *p*-value = $5.986\times 10^{-9}$), indicating a consistent and statistically robust improvement when gyroscope data are incorporated. IMU location (F = 23.39, *p*-value = $2.861\times 10^{-6}$) and classification model (F = 33.9, *p*-value = $1.298\times 10^{-8}$) also significantly contribute to performance variability. Among interaction terms, the IMU location × Num. classes interaction is particularly relevant (F = 23.18, *p*-value = $3.068\times 10^{-6}$), suggesting that the effect of sensor placement depends on classification granularity. In contrast, the Sensor Configuration × Num. Classes interaction is not statistically significant (*p*-value = 0.113), indicating that the benefit of including gyroscope signals is relatively stable across both fine- and coarse-grained tasks. The small residual error (23) suggests that the experimental design captures most of the variability in the F1-score.

Figure 4 provides a visual representation of these main effects through boxplot comparisons. A clear separation between the 4-intensity and 15-class conditions is observed, with the former consistently achieving higher median F1-scores and reduced dispersion. The inclusion of gyroscope data produces a systematic rightward shift in the F1-score distribution, confirming that performance gains are not model-specific but structurally consistent across configurations. With respect to IMU location, wrist-mounted sensors tend to achieve higher medians in the 15-class task, whereas thigh-mounted sensors show superior and more stable performance in the 4-class intensity classification (see Figure 6). Model-wise, Random Forest and XGBoost exhibit compact and consistently high distributions, while CNN ESANN shows greater variability, particularly under accelerometer-only configurations. CNN CAPTURE-24 demonstrates competitive performance, especially when gyroscope information is included, reinforcing the statistical findings from Table 3.

The aggregated confusion matrix (Figure 5) further elucidates class-level behavior under a representative high-performing configuration. For this experiment, the mapping was defined as follows: walking-related activities (including walking with groceries, walking while using a phone or reading a book, walking at usual speed, and walking in a zigzag pattern) were grouped under the walking superclass. Household activities such as standing, sweeping, folding towels, or moving books were categorized as household chores. Standing while using a computer was classified as standing. Rest periods labeled as resting phase with K5 were grouped under sleep. Incremental cycling on an ergometer was classified as bicycling. Sitting-related activities, including reading, using a computer, or watching TV, were grouped under sitting. More dynamic movements, such as ascending and descending stairs, were categorized as mixed activity. Finally, running at self-selected speed (20 m walkway) was classified as sports. The strong diagonal dominance (values ranging from 0.93 to 1.00) indicates high within-class accuracy for the aggregated ‘superclasses’. Misclassifications are concentrated between biomechanically related categories, such as sleep and sitting (sedentary postures), standing and household chores (low-intensity upright activities), and walking and mixed activity (locomotor patterns with overlapping kinematic signatures). This structured error pattern suggests that misclassifications arise from genuine biomechanical similarity rather than random model instability.

**Figure 7:**
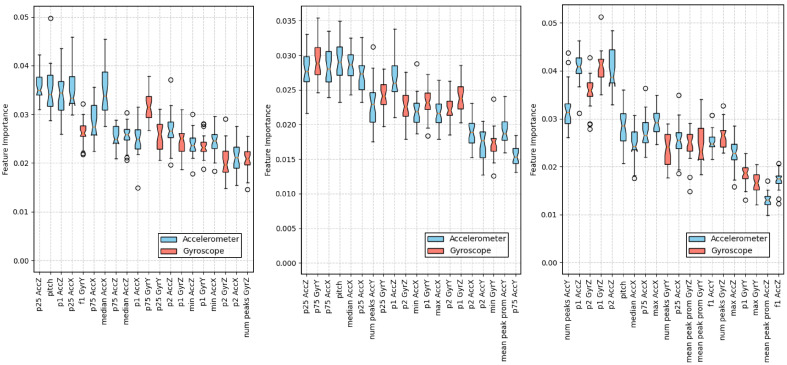
Distribution of the 20 most important features at each sensor location (thigh–left, wrist–center, and hip–right), obtained from a Random Forest classifier trained on the 15 laboratory-controlled activity classes and evaluated over 30 independent runs. Feature importance corresponds to the normalized mean decrease in impurity associated with each descriptor. Boxplots summarize the variability of feature importance across runs, with accelerometer-derived features shown in blue and gyroscope-derived features shown in red. Feature labels on the *x*-axis represent statistical descriptors extracted from 10-s windows of inertial signals. Subscripts *x*, *y*, and *z* denote the sensor axes in the local coordinate frame, while the prefixes Acc and Gyro refer to linear acceleration and angular velocity signals, respectively. The displayed descriptors include time-domain statistics (e.g., percentiles $p_{25}$ and $p_{75}$, median, minimum (min), and maximum (max)), peak-related measures (e.g., num. peaks detected), frequency-domain characteristics (e.g., power ($p_{x}$) and frequency ($f_{x}$) of the $x_{th}$ harmonics), and orientation-related angular variables (roll, pitch, and yaw) derived from accelerometer/gyroscope data.

For the feature importance analysis, in the case of the thigh-mounted wearable sensor, 40.00% of the 40 features identified as relevant contain gyroscope-related information. A similar analysis for the wrist-mounted wearable sensor showed that 39.53% of the 43 relevant features are derived from gyroscope data. Finally, for the hip-mounted wearable sensor, 46.34% of the 41 features considered relevant include information associated with the gyroscope (see an example in Figure 7).

## 4. Discussion

The analysis presented in this study is based on data from the WearablePerMed project, an initiative aligned with the IMPaCT cohort in terms of general methodology, monitor model (i.e., MATRIX 003) and programming parameters (i.e., 25 Hz), sensor placement (i.e., wrist and thigh) and the information collected (i.e., accelerometer and gyroscope), enabling a systematic evaluation of controlled laboratory-based activities classification from inertial sensors. The use of this single, purpose-oriented, and carefully curated dataset allows (i) the generation of methodological knowledge that is directly relevant and applicable to the ongoing IMPaCT cohort, and (ii) the isolation of the main effects under study without confounding factors arising from differences in hardware, sampling frequency, annotation protocols, or participant populations.

Four key factors were investigated: classification granularity (15 laboratory-controlled activities and 4 activity intensity levels), sensor placement (thigh, hip, wrist), classification model (Random Forest, XGBoost, Convolutional Neural Networks), and sensor configuration (accelerometer only and accelerometer + gyroscope). Evaluating these factors in combination provides evidence on their relative contributions to classification performance and establishes important insights for wearable-based activity recognition in large-scale cohort studies. Beyond capturing the natural heterogeneity in participants’ movement patterns, our results offer practical guidance for future research within the IMPaCT cohort and for similar population-based studies.

We explored two complementary approaches to activity classification. First, we aimed to distinguish all individual activities in the dataset to achieve the highest level of granularity or detail. Second, we approached classification from the perspective of energy expenditure, grouping activities according to their intensity into sedentary, light, moderate, and vigorous categories.

For fine-grained classification (see Figure 5a), the average F1-score across classification models, sensor configurations, and sensor locations was 62.60%. Confusion (see Figure 5a) primarily occurs between very similar activities, such as walking with groceries, walking while using a phone or reading a book, walking at a normal pace, and walking in a zigzag pattern. The subtle differences among these activities make it difficult for the models to distinguish them, leading to frequent misclassifications and lowering the overall F1-score.

For the coarse-grained classification (see Figure 6) into the four activity intensity levels, the averaged F1-score increased to 76.44%. In this scenario, all model families effectively discriminated between moderate and vigorous activity levels. However, some confusion occurred between light and moderate levels, which can be attributed to how similar activities were assigned to different intensity categories in our experimental design. For example (see Table 1), walking while carrying groceries (4.8 METs) was assigned to the moderate activity intensity class, whereas walking while holding a phone or book (2.0 METs) was assigned to the light activity intensity class. This splitting of similar (i.e., walking-related) activities across intensity categories contributed to residual misclassification and a slightly lower overall F1-score.

These results highlight that classification performance is strongly dependent on the definition of target classes. While no universally accepted hierarchy of activity classes has yet been established, it appears that defining intermediate superclasses may provide a more meaningful and robust framework for evaluating model performance. Thus, as an exploratory analysis, we conducted an additional experiment mapping the 15 laboratory classes to the eight superclasses proposed in CAPTURE-24 [6]. This approach substantially increased the mean F1-score to 95.87% (in one of the 30 experiments carried out) for the Random Forest model using the thigh-mounted wearable sensor (see Figure 5b), highlighting the potential of superclass-based definitions for improving classification performance, while also emphasizing that performance metrics should be interpreted relative to class granularity. It is noteworthy that the sport activity achieves a classification accuracy of 100%. This result is likely related to the distinctive and highly dynamic movement patterns associated with this activity under controlled laboratory conditions, as well as to the fact that the “sport” class corresponds to a single, well-defined activity. In contrast, other classes, such as walking-related activities, group multiple sub-activities and therefore exhibit higher intra-class variability, which makes classification more challenging. Nevertheless, this result is specific to the experimental protocol and class definition considered, and generalisation or broader conclusions should be approached with caution. In this context, careful selection and justification of target class definitions are necessary to ensure meaningful evaluation in activity recognition studies, underscoring the importance of selecting and analyzing target classes appropriately for each specific problem.

Similarly, the XGBoost model in [6] reported an F1-score of 0.399 for 10 activity classes and 0.693 for four intensity-based superclasses. In comparison, our XGBoost implementation achieves an F1-score of 0.6652 for 15 activity classes and 0.8013 for four intensity superclasses, indicating improved performance. It is worth noting that the data in [6] were collected under free-living conditions and subsequently annotated by expert visual inspection, which may result in a less finely curated dataset than our laboratory-based dataset. The controlled execution of laboratory activities and the standardized labeling protocol adopted in the present study may partly explain the higher classification performance observed.

The mean F1-scores across classification granularity, classification model, and sensor configuration for each sensor location were 72.52% for the wrist, 70.89% for the thigh, and 65.16% for the hip. Overall, the wrist demonstrated the highest performance for laboratory-controlled activity classification, performing comparably to the thigh, whereas the hip showed the lowest performance. The superior performance of the wrist, in line with previous reports in the literature [11], may be explained by the fact that lower-body activities generate characteristic movement patterns that are indirectly reflected in wrist motion, while upper-body movements are not fully captured by thigh- or hip-mounted sensors. Additionally, the controlled laboratory setting of our study ensured relatively homogeneous wrist movement patterns, limiting the variability associated with context- and individual-dependent movement patterns (e.g., walking with hands in pockets), which are more common in free-living conditions and may challenge activity recognition models.

CNN models trained on thigh-mounted sensors with both an accelerometer and gyroscope, such as CNN CAPTURE-24, achieved an average F1 score of 69.84% for the 15 laboratory-controlled activities, outperforming the maximum of 69.46% reported in [13]. Compared with previous studies using public accelerometry datasets [11], fine-grained classification performance for thigh-mounted sensors in our study was slightly lower. Specifically, ref. [11] reported an accuracy of 0.6868 for thigh-mounted sensors using the same baseline CNN architecture, whereas the CNN ESANN model employed here, trained on the full set of thigh-mounted accelerometer and gyroscope signals, achieved an average F1-score of 0.4520. This lower performance can be attributed to the strict separation of participants between the training and test sets adopted in the present study, which was not enforced in [11], potentially leading to overestimated generalization in that previous work. In contrast, wrist-mounted data classified with CNN ESANN model reached an F1-score of 0.7094 in the present study, exceeding the 0.6788 reported in [11]. This improvement can be attributed to the reduced variability of wrist movements during controlled laboratory activities, recorded under a rigid protocol that limits context- or person-dependent movement variations more common in free-living conditions.

Incorporating gyroscope data enhances classification performance, as evidenced by ANOVA comparisons. When using only accelerometer data, the mean F1-score across classification granularity, classification model and sensor location was 65.39%, whereas combining accelerometer and gyroscope data increased the mean F1-score to 73.65%, representing an improvement of approximately 8.3 percentage points. This performance gain is further supported by feature importance analysis, which shows that gyroscope-related features account for roughly 40% of the most informative predictors.

It is important, however, to contextualise this improvement. While the inclusion of gyroscope data improves performance, the decision to incorporate a gyroscope should balance the benefits in classification accuracy against the trade-offs in terms of device additional cost, complexity, and energy consumption associated with the sensor. For example, a previous work showed that the gyroscope consumes at least six times more energy than the accelerometer [14], whereas gyro-free models can reduce the economic cost of the inertial measurement devices [15,16].

We adopted state-of-the-art models commonly used in similar population-based studies, including decision tree-based approaches under bagging and boosting frameworks (i.e., Random Forest and XGBoost), as well as convolutional neural networks (i.e., CNN ESANN and CNN CAPTURE-24). Figure 4 summarises the distribution of classification performance across experimental configurations using boxplots, while numerical mean F1-scores are reported here to facilitate interpretation and comparison. For the fine-grained classification task (15 laboratory-controlled activity classes), the averaged F1-scores across sensor location and sensor configuration were 51.75% for CNN ESANN, 64.35% for CNN CAPTURE-24, 67.79% for Random Forest, and 66.52% for XGBoost, with Random Forest achieving the highest accuracy. In the coarse-grained classification (4 activity intensity levels), the averaged F1-scores were 72.17% for CNN ESANN, 74.47% for CNN CAPTURE-24, 79.00% for Random Forest and 80.13% for XGBoost, with the last performing best.

Nonetheless, despite these differences in performance metrics, the models show no substantial disparities in practical performance. CNN ESANN, a simplified convolutional architecture, exhibits slightly lower accuracy, but CNN CAPTURE-24, Random Forest, and XGBoost quantitively perform comparably, with only minor variations in averaged F1-scores.

Thus, although these differences may reach statistical significance, they are unlikely to be practically meaningful, as other factors such as classification granularity, sensor location, and sensor configuration exert a far greater impact on performance. Overall, these findings indicate that all evaluated models provide broadly comparable performance for both fine- and coarse-grained activity classification, emphasizing the importance of prioritizing the most influential experimental factors when interpreting results.

The results obtained in this study are comparable to or exceed previously reported performance in physical activity classification. For instance, the Random Forest model presented in [6], based on wrist accelerometry, achieves an average F1-score of 0.388 for 10 activity classes, whereas our implementation reached 0.6779 for a more challenging task involving 15 activity classes.

## 5. Limitations of the Study

Signal patterns were visually inspected to identify segmentation markers, which may have introduced minor inaccuracies and resulted in a small number of mislabeled windows within the dataset. Additionally, some activities exhibited brief periods of inactivity for a few participants, possibly due to interruptions during the task execution, which could not be removed, occasionally leading to incorrectly labeled segments. However, the impact of these issues on overall classification performance is expected to be minimal.

The activity classification models developed in this study were evaluated using data collected under controlled laboratory conditions. Consequently, when applying these models in real-world settings, performance levels may differ from those observed in this study. The findings should therefore be interpreted as upper-bound estimates obtained under controlled conditions.

This research is specifically oriented toward the classification of activities of daily living (ADL), which are the primary target in population-based health cohorts. The recognition of other activity domains, such as sports or occupational tasks, was beyond the scope of this study.

Sensor laterality effects were not explicitly modelled or corrected. Sensors were placed according to the non-dominant wrist and contralateral thigh following the IMPaCT cohort protocol, reflecting real deployment conditions, but no left/right normalization was applied. Although this is unlikely to affect the relative comparisons presented in this work, laterality may play a role in classification performance.

## 6. Future Work

The first line of future work involves systematically applying a time-synchronisation method to accelerometry signals from different inertial sensors; this would enable the exploration of classification models that combine information from different body locations simultaneously. Furthermore, utilising unstructured activity (free-living conditions), multi-dataset scenarios, and more dynamic activity domains during the training of classification models will enhance their ability to generalise to activities of daily living, beyond controlled laboratory conditions.

Motivated by the observed differences in classification performance between the 4-class target (activity intensity levels) and the 15-class target, as well as by the exploratory encouraging results obtained using an intermediate 8-class definition, future work could explore the definition of a representative and well-structured set of target activity classes. Such efforts should aim to balance activity coverage, interpretability, and discriminability, in order to capture relevant activity patterns while enabling accurate and robust classification. Identifying suitable class taxonomies for this purpose remains an important topic for further investigation.

Validation in fully free-living real-world scenarios represents a relevant and necessary direction for future research, as the classification models are ultimately intended for the analysis of habitual free-living physical activity and should be assessed under unconstrained conditions.

Future work could examine the effect of sensor laterality, for example by assessing whether left/right normalization strategies influence activity recognition performance.

## 7. Conclusions

The present study provides evidence that incorporating gyroscope-derived angular velocity signals enhances physical activity classification using wearable sensors, although the improvement over accelerometer-only models is moderate. Across all single-sensor placements evaluated, models using both accelerometry and gyroscope data generally outperformed accelerometer-only models in terms of F1-score and overall accuracy. Regarding sensor location, performance varied by task granularity: for fine-grained activity classification, the wrist generally achieved the highest performance, while for coarse-grained intensity-level classification, the thigh consistently outperformed other locations.

The addition of gyroscope data is particularly valuable for capturing subtle motion patterns not fully represented by linear acceleration, thereby slightly reducing intra- and inter-participant variability in activity classification. These findings were consistent across multiple machine learning and deep learning models, including Random Forest, XGBoost, and CNNs. Therefore, multimodal sensing with accelerometers and gyroscopes is recommended for future population cohort studies, as it provides a modest but meaningful improvement in classification fidelity while maintaining feasible sensor configurations.

## Figures and Tables

**Table 1:** Creation of activity intensity classes based on metabolic equivalent of task (MET) values from the Compendium of Physical Activities (CPA) [9,10].

Specific Activity	METs	CPA Code	Sed.	LI	MI	VI
Resting phase with K5	1.3	7026	✓			
Sitting watching TV	1.3	9030	✓			
Sitting reading	1.3	9030	✓			
Sitting using computer	1.0	9045	✓			
Standing using computer	1.8	9070		✓		
Standing folding towels	2.0	5090		✓		
Walking with phone or book	2.0	17150		✓		
Walking zigzag	2.0	17150		✓		
Standing moving books	5.8	5120			✓	
Standing sweeping	3.3	5010			✓	
Walking with groceries	4.8	17260			✓	
Walking at usual speed	3.5	17250			✓	
Climbing and descending stairs	3.5	17070			✓	
Running	7.0	12020				✓
Incremental cycle ergometer	10.0	12180				✓

Abbreviations: Sed., sedentary activity (<1.5 METs); LI, light-intensity activity (1.5 to <3.0 METs); MI, moderate-intensity activity (3.0 to <6.0 METs); VI, vigorous-intensity activity (≥6.0 METs).

**Table 2:** Summary of classification results across classification models, wearable sensor locations, sensor configurations, and class granularity. T denotes *Thigh*, W denotes *Wrist*, H denotes *Hip*. Acc denotes *Accelerometer* and Acc + Gyro denotes *Accelerometer and Gyroscope*. The # symbol refers to the *number of*, in this case, *physical activity classes*.

Classification Model	Sensor Location	# Classes	Sensor Configuration	F1-Score (Mean ± STD)
Random Forest	T	15 (Laboratory-controlled activities)	Acc	62.48±2.19
Random Forest	W	15 (Laboratory-controlled activities)	Acc	71.51±3.07
Random Forest	H	15 (Laboratory-controlled activities)	Acc	61.92±2.64
Random Forest	T	15 (Laboratory-controlled activities)	Acc + Gyro	66.43±2.27
Random Forest	W	15 (Laboratory-controlled activities)	Acc + Gyro	75.38±2.60
Random Forest	H	15 (Laboratory-controlled activities)	Acc + Gyro	69.04±2.35
Random Forest	T	4 (Activity intensity)	Acc	80.14±1.29
Random Forest	W	4 (Activity intensity)	Acc	78.37±1.75
Random Forest	H	4 (Activity intensity)	Acc	73.22±1.79
Random Forest	T	4 (Activity intensity)	Acc + Gyro	83.46±1.54
Random Forest	W	4 (Activity intensity)	Acc + Gyro	80.27±1.41
Random Forest	H	4 (Activity intensity)	Acc + Gyro	78.51±1.65
XGBoost	T	15 (Laboratory-controlled activities)	Acc	62.99±3.92
XGBoost	W	15 (Laboratory-controlled activities)	Acc	71.86±3.90
XGBoost	H	15 (Laboratory-controlled activities)	Acc	55.23±3.10
XGBoost	T	15 (Laboratory-controlled activities)	Acc + Gyro	65.36±3.12
XGBoost	W	15 (Laboratory-controlled activities)	Acc + Gyro	75.45±3.57
XGBoost	H	15 (Laboratory-controlled activities)	Acc + Gyro	68.20±4.01
XGBoost	T	4 (Activity intensity)	Acc	81.77±2.45
XGBoost	W	4 (Activity intensity)	Acc	78.45±2.72
XGBoost	H	4 (Activity intensity)	Acc	77.06±2.13
XGBoost	T	4 (Activity intensity)	Acc + Gyro	83.52±2.01
XGBoost	W	4 (Activity intensity)	Acc + Gyro	81.05±2.04
XGBoost	H	4 (Activity intensity)	Acc + Gyro	78.94±2.17
CNN ESANN	T	15 (Laboratory-controlled activities)	Acc	43.60±2.10
CNN ESANN	W	15 (Laboratory-controlled activities)	Acc	49.11±5.51
CNN ESANN	H	15 (Laboratory-controlled activities)	Acc	40.69±5.41
CNN ESANN	T	15 (Laboratory-controlled activities)	Acc + Gyro	45.20±4.68
CNN ESANN	W	15 (Laboratory-controlled activities)	Acc + Gyro	70.94±4.12
CNN ESANN	H	15 (Laboratory-controlled activities)	Acc + Gyro	60.98±5.76
CNN ESANN	T	4 (Activity intensity)	Acc	76.08±3.48
CNN ESANN	W	4 (Activity intensity)	Acc	64.93±3.77
CNN ESANN	H	4 (Activity intensity)	Acc	61.87±10.98
CNN ESANN	T	4 (Activity intensity)	Acc + Gyro	81.77±2.17
CNN ESANN	W	4 (Activity intensity)	Acc + Gyro	77.94±2.76
CNN ESANN	H	4 (Activity intensity)	Acc + Gyro	70.42±10.66
CNN CAPTURE-24	T	15 (Laboratory-controlled activities)	Acc	62.49±6.05
CNN CAPTURE-24	W	15 (Laboratory-controlled activities)	Acc	61.12±4.33
CNN CAPTURE-24	H	15 (Laboratory-controlled activities)	Acc	49.56±6.42
CNN CAPTURE-24	T	15 (Laboratory-controlled activities)	Acc + Gyro	69.84±6.14
CNN CAPTURE-24	W	15 (Laboratory-controlled activities)	Acc + Gyro	76.80±5.82
CNN CAPTURE-24	H	15 (Laboratory-controlled activities)	Acc + Gyro	66.27±6.34
CNN CAPTURE-24	T	4 (Activity intensity)	Acc	82.43±4.43
CNN CAPTURE-24	W	4 (Activity intensity)	Acc	66.22±5.25
CNN CAPTURE-24	H	4 (Activity intensity)	Acc	56.34±5.60
CNN CAPTURE-24	T	4 (Activity intensity)	Acc + Gyro	86.60±2.33
CNN CAPTURE-24	W	4 (Activity intensity)	Acc + Gyro	80.93±5.65
CNN CAPTURE-24	H	4 (Activity intensity)	Acc + Gyro	74.29±4.13

**Table 3:** Analysis of variance (ANOVA) of the F1-score.

Source	Sum of Squares	d.f.	Mean Square	F-Value	*p*-Value
Model	1039.54	3	346.51	33.90	$1.30\times 10^{-8}$
Wearable sensor	478.20	2	239.10	23.39	$2.86\times 10^{-6}$
Sensor configuration	817.99	1	817.99	80.03	$5.99\times 10^{-9}$
NumClasses	2298.13	1	2298.13	224.85	$2.30\times 10^{-13}$
Model × Wearable sensor	184.07	6	30.68	3.00	$2.58\times 10^{-2}$
Model × Sensor configuration	197.05	3	65.68	6.43	$2.54\times 10^{-3}$
Model × NumClasses	192.20	3	64.07	6.27	$2.88\times 10^{-3}$
Wearable sensor × Sensor configuration	126.24	2	63.12	6.18	$7.13\times 10^{-3}$
Wearable sensor × NumClasses	473.87	2	236.94	23.18	$3.07\times 10^{-6}$
Sensor configuration × NumClasses	27.77	1	27.77	2.72	$1.13\times 10^{-1}$
Error	235.08	23	10.22		
Total	6070.14	47			

## Data Availability

The dataset used in this study can be shared upon reasonable request to the authors.
